# Robust Z-Estimators for Semiparametric Moment Condition Models

**DOI:** 10.3390/e25071013

**Published:** 2023-06-30

**Authors:** Aida Toma

**Affiliations:** 1 Department of Applied Mathematics, Bucharest University of Economic Studies, 010374 Bucharest, Romania; aida.toma@csie.ase.ro; 2 “Gheorghe Mihoc-Caius Iacob” Institute of Mathematical Statistics and Applied Mathematics of the Romanian Academy, 050711 Bucharest, Romania

**Keywords:** moment condition models, divergences, robustness

## Abstract

In the present paper, we introduce a class of robust Z-estimators for moment condition models. These new estimators can be seen as robust alternatives for the minimum empirical divergence estimators. By using the multidimensional Huber function, we first define robust estimators of the element that realizes the supremum in the dual form of the divergence. A linear relationship between the influence function of a minimum empirical divergence estimator and the influence function of the estimator of the element that realizes the supremum in the dual form of the divergence led to the idea of defining new Z-estimators for the parameter of the model, by using robust estimators in the dual form of the divergence. The asymptotic properties of the proposed estimators were proven, including here the consistency and their asymptotic normality. Then, the influence functions of the estimators were derived, and their robustness is demonstrated.

## 1. Introduction

A moment condition model is a family $M^{1}$ of probability measures, all defined on the same measurable space $\left(R^{m},B\left(R^{m}\right)\right)$, such that

(1) $$\int g\left(x,\theta \right)dQ\left(x\right)=0,\;\;\mathrm{for}\;\mathrm{all}\;\;Q\in M^{1}.$$

The parameter θ belongs to $\Theta \subset R^{d}$; the function $g:={\left(g_{1},\ldots ,g_{l}\right)}^{⊤}$ is defined on $R^{m}\times \Theta$, each of the $g_{i}$’s being real-valued, l≥d, and the functions $g_{1},\ldots ,g_{l}$ and $1_{X}$ are supposed to be linearly independent. Denote by $M^{1}$ the set of all probability measures on $\left(R^{m},B\left(R^{m}\right)\right)$ and

(2) $$M_{\theta }^{1}:=\left\{Q\in M^{1}:\int g\left(x,\theta \right)dQ\left(x\right)=0\right\},$$

such that

(3) $$M^{1}=\underset{\theta \in \Theta }{⋃}M_{\theta }^{1}.$$

Let $X_{1},\cdots ,X_{n}$ be an i.i.d. sample on the random vector *X* with unknown probability distribution $P_{0}$. We considered the problem of the estimation of the parameter $\theta_{0}$ for which the constraints of the model are satisfied:

(4) $$\int g\left(x,\theta_{0}\right)dP_{0}\left(x\right)=0.$$

We supposed that $\theta_{0}$ is the unique solution of Equation (4). Thus, we assumed that information about $\theta_{0}$ and $P_{0}$ is available in the form of l≥d functionally independent unbiased estimating functions, and we used this information to estimate $\theta_{0}$.

Among the best-known estimation methods for moment condition models, we mention the generalized method of moments (GMM) [1], the continuous updating (CU) estimator [2], the empirical likelihood (EL) estimator [3,4], the exponential tilting (ET) estimator [5], and the generalized empirical likelihood (GEL) estimators [6]. Although the EL estimator is superior to other estimators in terms of higher-order asymptotic properties, these properties hold only under the correct specification of the moment conditions. In [7] was proposed the exponentially tilted empirical likelihood (ETEL) estimator, which has the same higher-order property as the EL estimator under the correct specification, while maintaining the usual asymptotic properties such as the consistency and asymptotic normality under misspecification. The so-called information and entropy econometric techniques have been proposed to improve the finite sample performance of the GMM-estimators and tests (see, e.g., [4,5]).

Some recent methods for the estimation and testing of moment condition models are based on using divergences. Divergences between probability measures are widely used in statistics and data science in order to perform inference in models of various kinds, parametric or semiparametric. Statistical methods based on divergence minimization extend the likelihood paradigm and often have the advantage of providing a trade-off between efficiency and robustness [8,9,10,11]. A general methodology for the estimation and testing of moment condition models was developed in [12]. This approach is based on minimizing divergences in their dual form and allows the asymptotic study of the estimators, called minimum empirical divergence estimators, and of the associated test statistics, both under the model and under misspecification of the model. The approach based on minimizing dual forms of divergences was initially used in the case of parametric models, the results being published in a series of articles [13,14,15,16]. The broad class of minimum empirical divergence estimators contains in particular the EL estimator, the CU estimator, as well as the ET estimator mentioned above. Using the influence function as the robustness measure, it has been shown that the minimum empirical divergence estimators are not robust, because the corresponding influence functions are generally not bounded [17]. On the other hand, the minimum empirical divergence estimators have the same efficiency of first order, and moreover, the EL estimator, which belong to this class, is superior in higher-order efficiency. Therefore, proposing robust versions of the minimum empirical divergence estimators would bring a trade-off between robustness and efficiency. These aspects motivated our studies in the present paper.

Some robust estimation methods for moment condition models have been proposed in the literature, for example in [18,19,20,21,22]. In the present paper, we introduce a class of robust Z-estimators for moment condition models. These new estimators can be seen as robust alternatives for the minimum empirical divergence estimators. By using the multidimensional Huber function, we first define robust estimators of the element that realizes the supremum in the dual form of the divergence. A linear relationship between the influence function of a minimum empirical divergence estimator and the influence function of the estimator of the element that realizes the supremum in the dual form of the divergence led to the idea of defining new Z-estimators for the parameter of the model, by using robust estimators in the dual form of the divergence. The asymptotic properties of the proposed estimators were proven, including here the consistency and their asymptotic normality. Then, the influence functions of the estimators were derived, and their robustness is demonstrated.

The paper is organized as follows. In Section 2, we briefly recall the context and the definitions of the minimum empirical divergence estimators, these being necessary for defining the new estimators. In Section 3, the new Z-estimators for moment condition models are defined. The asymptotic properties of these estimators were proven, including here the consistency and their asymptotic normality. Then, the influence functions of the estimators were derived, and their robustness is demonstrated. The proofs of the theoretical results are deferred in Appendix A.

## 2. Minimum Empirical Divergence Estimators

### 2.1. Statistical Divergences

Let φ be a convex function defined on R and $\left[0,\infty \right]$-valued, such that φ(1)=0, and let $P\in M^{1}$ be some probability measure. For any signed finite measure *Q* defined on the same measurable space $\left(R^{m},B\left(R^{m}\right)\right)$, absolutely continuous (a.c.) with respect to *P*, the φ divergence between *Q* and *P* is defined by

(5) $$D_{\varphi }\left(Q,P\right):=\int \varphi \left(\frac{dQ}{dP}\left(x\right)\right)dP\left(x\right).$$

When *Q* is not a.c. with respect to P, we set $D_{\varphi }\left(Q,P\right)=\infty$. This extension, for the case when *Q* is not absolutely continuous with respect to *P*, was considered in order to have a unique definition of divergences, appropriate for both cases—that of continuous probability laws and that of discrete probability laws.

This definition extends the one of divergences between probability measures [23], and the necessity of working with signed finite measures will be explained in Section 2.2.

Largely used in information theory, the Kullback–Leibler divergence is associated with the real convex function φ(x):=xlogx−x+1 and is defined by

$$KL\left(Q,P\right):=\int \log\left(\frac{dQ}{dP}\right)dQ.$$

The modified Kullback–Leibler divergence is associated with the convex function φ(x):=−logx+x−1 and is defined through

$$KL_{m}\left(Q,P\right):=\int -\log\left(\frac{dQ}{dP}\right)dP.$$

Other divergences, largely used in inferential statistics, are the $\chi^{2}$ and the modified $\chi^{2}$ divergences, namely

$$\chi^{2}\left(Q,P\right):=\frac{1}{2}\int {\left(\frac{dQ}{dP}-1\right)}^{2}dP,$$

$$\chi_{m}^{2}\left(Q,P\right):=\frac{1}{2}\int \frac{{\left(\frac{dQ}{dP}-1\right)}^{2}}{\frac{dQ}{dP}}dP,$$

these being associated with the convex functions $\varphi \left(x\right):=\frac{1}{2}{\left(x-1\right)}^{2}$ and $\varphi \left(x\right):=\frac{1}{2}{\left(x-1\right)}^{2}/x$, respectively. The Hellinger distance and the $L_{1}$ distance are also φ divergences. They are associated with the convex functions $\varphi \left(x\right):=2{\left(\sqrt{x}-1\right)}^{2}$ and φ(x):=|x−1|, respectively.

All the preceding examples, except the $L_{1}$ distance, belong to the class of power divergences introduced by Cressie and Read [24] and defined by the convex functions:

(6) $$x\in R_{+}^{*}\mapsto \varphi_{\gamma }\left(x\right):=\frac{x^{\gamma }-\gamma x+\gamma -1}{\gamma (\gamma -1)},$$

for γ∈R∖{0,1} and $\varphi_{0}\left(x\right):=-\log x+x-1$, $\varphi_{1}\left(x\right):=x\log x-x+1$. The Kullback–Leibler divergence is associated with $\varphi_{1}$, the modified Kullback–Leibler with $\varphi_{0}$, the $\chi^{2}$ divergence with $\varphi_{2}$, the modified $\chi^{2}$ divergence with $\varphi_{-1}$, and the Hellinger distance with $\varphi_{1/2}$. When $\varphi_{\gamma }$ is not defined on (−∞,0) or when $\varphi_{\gamma }$ is not convex, the definition of the corresponding power divergence function $Q\in M^{1}\mapsto D_{\varphi_{\gamma }}\left(Q,P\right)$ can be extended to the whole set of signed finite measures by taking the following extension of $\varphi_{\gamma }$:

$$\varphi_{\gamma }:\;x\in R\mapsto \varphi_{\gamma }\left(x\right)1_{\left[0,\infty \right)}\left(x\right)+\left(+\infty \right)1_{\left(-\infty ,0\right)}\left(x\right).$$

The φ divergence between some set Ω of signed finite measures and a probability measure *P* is defined by

(7) $$D_{\varphi }\left(\Omega ,P\right)=\underset{Q\in \Omega }{\inf}D_{\varphi }\left(Q,P\right).$$

Assuming that $D_{\varphi }\left(\Omega ,P\right)$ is finite, a measure $Q^{*}\in \Omega$ is called a φ-projection of *P* on Ω if

$$D_{\varphi }\left(Q^{*},P\right)\leq D_{\varphi }\left(Q,P\right),\;\mathrm{for}\;\mathrm{all}\;Q\in \Omega .$$

### 2.2. Minimum Empirical Divergence Estimators

Let $X_{1},\cdots ,X_{n}$ be an i.i.d. sample on the random vector *X* with the probability distribution $P_{0}$. The “plug-in” estimator of the φ divergence $D_{\varphi }\left(M_{\theta }^{1},P_{0}\right)$ between the set $M_{\theta }^{1}$ and the probability measure $P_{0}$ is defined by replacing $P_{0}$ with the empirical measure associated with the sample. More precisely,

(8) $${\hat{D}}_{\varphi }\left(M_{\theta }^{1},P_{0}\right)=\underset{Q\in M_{\theta }^{1}}{\inf}D_{\varphi }\left(Q,P_{n}\right)=\underset{Q\in M_{\theta }^{1}}{\inf}\int \varphi \left(\frac{dQ}{dP_{n}}\left(x\right)\right)dP_{n}\left(x\right),$$

where $P_{n}:=\frac{1}{n}\sum_{i=1}^{n}\delta_{X_{i}}$ is the empirical measure associated with the sample, $\delta_{x}$ being the Dirac measure putting all mass at *x*. If the projection of the measure $P_{n}$ on $M_{\theta }^{1}$ exists, it is a law a.c. with respect to $P_{n}$. Then, it is natural to consider

(9) $$M_{\theta }^{\left(n\right)}=\left\{Q\in M^{1}:Q\;a.c.\;\mathrm{with}\;\mathrm{respect}\;\mathrm{to}\;P_{n}\;\mathrm{and}\;\sum_{i=1}^{n}g\left(X_{i},\theta \right)Q\left(X_{i}\right)=0\right\},$$

and then, the plug-in estimator (8) can be written as

(10) $${\hat{D}}_{\varphi }\left(M_{\theta }^{1},P_{0}\right)=\underset{Q\in M_{\theta }^{\left(n\right)}}{\inf}\frac{1}{n}\sum_{i=1}^{n}\varphi \left(nQ\left(X_{i}\right)\right).$$

The infimum in the above expression (10) may be achieved at a point situated on the frontier of the set $M_{\theta }^{\left(n\right)}$, a case in which the Lagrange method for characterizing the infimum and computing ${\hat{D}}_{\varphi }\left(M_{\theta }^{1},P_{0}\right)$ cannot be applied. In order to avoid this difficulty, Broniatowski and Keziou [12,25] proposed to work on sets of signed finite measures and defined

(11) $$M_{\theta }:=\left\{Q\in M:\int dQ=1\;\mathrm{and}\;\int g\left(x,\theta \right)dQ\left(x\right)=0\right\},$$

where *M* denotes the set of all signed finite measures on the measurable space $\left(R^{m},B\left(R^{m}\right)\right)$, and

(12) $$M:=\underset{\theta \in \Theta }{⋃}M_{\theta }.$$

They showed that, if $Q_{1}^{*}$ the projection of $P_{n}$ on $M_{\theta }^{1}$ is an interior point of $M_{\theta }^{1}$ and $Q^{*}$ the projection of $P_{n}$ on $M_{\theta }$ is an interior point of $M_{\theta }$, then both approaches based on signed finite measures, respectively on probability measures, for defining minimum divergence estimators coincide. On the other hand, in the case when $Q_{1}^{*}$ is a frontier point of $M_{\theta }^{1}$, the estimator of the parameter $\theta_{0}$ defined using the context of signed finite measures converges to $\theta_{0}$. These aspects justify the substitution of $M_{\theta }^{1}$ by $M_{\theta }$.

In the following, we briefly recall the definitions of the estimators for the moment condition proposed in [12] in the context of signed finite measure sets.

Denote by $\bar{g}$ the function defined on $R^{m}\times \Theta$ and $R^{l+1}$-valued:

(13) $$\bar{g}\left(x,\theta \right):={\left(1_{X}\left(x\right),g_{1}\left(x,\theta \right),\ldots ,g_{l}\left(x,\theta \right)\right)}^{⊤}.$$

Given a φ divergence, when the function φ is strictly convex on its domain, denote

$$\varphi^{*}\left(u\right):=u{\varphi \prime }^{-1}\left(u\right)-\varphi \left({\varphi \prime }^{-1}\left(u\right)\right),$$

the convex conjugate of the function φ. For a given probability measure $P\in M^{1}$ and a fixed θ∈Θ, define

(14) $$\Lambda_{\theta }\left(P\right):=\{t\in R^{l+1}:\int |\varphi^{*}\left(t_{0}+\sum_{j=1}^{l}t_{j}g_{j}\left(x,\theta \right)\right)\left|dP\left(x\right)<\infty \right\}.$$

We also use the notations $\Lambda_{\theta }$ for $\Lambda_{\theta }\left(P_{0}\right)$ and $\Lambda_{\theta }^{\left(n\right)}$ for $\Lambda_{\theta }\left(P_{n}\right)$.

Supposing that $P_{0}$ admits a projection $Q_{\theta }^{*}$ on $M_{\theta }$ with the same support as $P_{0}$ and that the function φ is strictly convex on its domain, then the φ divergence $D_{\varphi }\left(M_{\theta },P_{0}\right)$ admits the dual representation:

(15) $$D_{\varphi }\left(M_{\theta },P_{0}\right)=\underset{t\in \Lambda_{\theta }}{\sup}\int m\left(x,\theta ,t\right)dP_{0}\left(x\right),$$

where $m\left(x,\theta ,t\right):=t_{0}-\varphi^{*}\left(t^{⊤}\bar{g}\left(x,\theta \right)\right).$

The supremum in (15) is unique and is reached at a point that we denote as $t_{\theta }=t_{\theta }\left(P_{0}\right)$:

(16) $$t_{\theta }:=\arg\underset{t\in \Lambda_{\theta }}{\sup}\int m\left(x,\theta ,t\right)dP_{0}\left(x\right).$$

Then, $D_{\varphi }\left(M_{\theta },P_{0}\right)$, $t_{\theta }$, $D_{\varphi }\left(M,P_{0}\right)$ and $\theta_{0}$ can be estimated respectively by

(17) $$\begin{array}{ccc} & & {\hat{D}}_{\varphi }\left(M_{\theta },P_{0}\right):=\underset{t\in \Lambda_{\theta }^{\left(n\right)}}{\sup}\int m\left(x,\theta ,t\right)dP_{n}\left(x\right), \end{array}$$

(18) $$\begin{array}{c} {\hat{t}}_{\theta }:=\arg\underset{t\in \Lambda_{\theta }^{\left(n\right)}}{\sup}\int m\left(x,\theta ,t\right)dP_{n}\left(x\right), \end{array}$$

(19) $$\begin{array}{ccc} & & {\hat{D}}_{\varphi }\left(M,P_{0}\right):=\underset{\theta \in \Theta }{\inf}\underset{t\in \Lambda_{\theta }^{\left(n\right)}}{\sup}\int m\left(x,\theta ,t\right)dP_{n}\left(x\right), \end{array}$$

(20) $$\begin{array}{ccc} & & {\hat{\theta }}_{\varphi }:=\arg\underset{\theta \in \Theta }{\inf}\underset{t\in \Lambda_{\theta }^{\left(n\right)}}{\sup}\int m\left(x,\theta ,t\right)dP_{n}\left(x\right). \end{array}$$

The estimators defined in (20) are called minimum empirical divergence estimators. We refer to [12] for the complete study of the existence and of the asymptotic properties of the above estimators.

The influence functions of these estimators and corresponding robustness properties were studied in [17]. According to those results, for θ∈Θ fixed, the influence function of the estimator ${\hat{t}}_{\theta }$ is given by

(21) $$\mathrm{IF}\left(x;t_{\theta },P_{0}\right)=-{\left[\int \frac{\partial^{2}}{\partial^{2}t}m\left(y,\theta ,t_{\theta }\left(P_{0}\right)\right)dP_{0}\left(y\right)\right]}^{-1}\frac{\partial }{\partial t}m\left(x,\theta ,t_{\theta }\left(P_{0}\right)\right),$$

where

(22) $$\frac{\partial }{\partial t}m\left(x,\theta ,t\right)={\left(1,0_{l}\right)}^{⊤}-{\varphi \prime }^{-1}\left(t^{⊤}\bar{g}\left(x,\theta \right)\right)\bar{g}\left(x,\theta \right),$$

(23) $$\frac{\partial^{2}}{\partial^{2}t}m\left(x,\theta ,t\right)=-\frac{1}{\varphi \prime \prime \left({\varphi \prime }^{-1}\left(t^{⊤}\bar{g}\left(x,\theta \right)\right)\right)}\bar{g}\left(x,\theta \right)\bar{g}{\left(x,\theta \right)}^{⊤},$$

with the particular case $\theta =\theta_{0}$:

(24) $$\mathrm{IF}\left(x;t_{\theta_{0}},P_{0}\right)=-\varphi \prime \prime \left(1\right){\left[\int \bar{g}\left(y,\theta_{0}\right)\bar{g}{\left(y,\theta_{0}\right)}^{⊤}dP_{0}\left(y\right)\right]}^{-1}{\left(0,g{\left(x,\theta_{0}\right)}^{⊤}\right)}^{⊤}.$$

On the other hand, the influence function of the estimator ${\hat{\theta }}_{\varphi }$ is given by

(25) $$\begin{array}{c} \mathrm{IF}\left(x;T_{\varphi },P_{0}\right)=-{\left\{{\left[\int \frac{\partial }{\partial \theta }g\left(y,\theta_{0}\right)dP_{0}\left(y\right)\right]}^{⊤}{\left[\int g\left(y,\theta_{0}\right)g{\left(y,\theta_{0}\right)}^{⊤}dP_{0}\left(y\right)\right]}^{-1}\int \frac{\partial }{\partial \theta }g\left(y,\theta_{0}\right)dP_{0}\left(y\right)\right\}}^{-1}\\ \cdot {\left[\int \frac{\partial }{\partial \theta }g\left(y,\theta_{0}\right)\;dP_{0}\left(y\right)\right]}^{⊤}{\left[\int g\left(y,\theta_{0}\right)g{\left(y,\theta_{0}\right)}^{⊤}dP_{0}\left(y\right)\right]}^{-1}g\left(x,\theta_{0}\right). \end{array}$$

Since the function x↦g(x,θ) is usually not bounded, for example, when we have linear constraints, the influence function $\mathrm{IF}(x;T_{\varphi },P_{0})$ is not bounded; therefore, the minimum empirical divergence estimators ${\hat{\theta }}_{\varphi }$ defined in (20) are generally not robust.

Through the calculations, it can be seen that there is a connection between the influence functions $\mathrm{IF}(x;t_{\theta_{0}},P_{0})$ and $\mathrm{IF}(x;T_{\varphi },P_{0})$, namely the relation

$${\left[\int \frac{\partial }{\partial \theta }\bar{g}\left(y,\theta_{0}\right)dP_{0}\left(y\right)\right]}^{⊤}\cdot \left\{\frac{\partial }{\partial \theta }t\left(\theta_{0},P_{0}\right)\mathrm{IF}\left(x;T_{\varphi },P_{0}\right)+\mathrm{IF}\left(x;t_{\theta_{0}},P_{0}\right)\right\}=0.$$

Since $\mathrm{IF}(x;T_{\varphi },P_{0})$ is linearly related to $\mathrm{IF}(x;t_{\theta_{0}},P_{0})$, using a robust estimator of $t_{\theta }=t_{\theta }\left(P_{0}\right)$ in the original duality Formula (15) would lead to a new robust estimator of $\theta_{0}$. This is the idea at the basis of our proposal in this paper, for constructing new robust estimators for moment condition models.

## 3. Robust Estimators for Moment Condition Models

### 3.1. Definitions of New Estimators

In this section, we define robust versions of the estimators ${\hat{t}}_{\theta }$ from (18) and robust versions of minimum empirical divergence estimators ${\hat{\theta }}_{\varphi }$ from (20). First, we define robust estimators of $t_{\theta }$, by using a truncated version of the function $x\mapsto \frac{\partial }{\partial t}m\left(x,\theta ,t\right)$, and then, we insert such a robust estimator in the estimating equation corresponding to the minimum empirical divergence estimator. The truncated function is based on the multidimensional Huber function and contains a shift vector $\tau_{\theta }$ and a scale matrix $A_{\theta }$ to calibrate $t_{\theta }$ and, thus, $t_{\theta }$, which realizes the supremum in the duality formula, will also be the solution of a new equation based on the new truncated function.

For simplicity, for fixed θ∈Θ, we also use the notation $m_{\theta }\left(x,t\right):=m\left(x,\theta ,t\right)$. With this notation, $t_{\theta }=t_{\theta }\left(P_{0}\right)$ defined in (16) is the unique solution of the equation:

(26) $$\int \frac{\partial }{\partial t}m_{\theta }\left(x,t_{\theta }\left(P_{0}\right)\right)dP_{0}\left(x\right)=0.$$

Consider the system

(27) $$\;\int \frac{\partial }{\partial t}m_{\theta }\left(y,t\right)dP_{0}\left(y\right)=0$$

(28) $$\;\int H_{c}\left(A\left[\frac{\partial }{\partial t}m_{\theta }\left(y,t\right)-\tau \right]\right)dP_{0}\left(y\right)=0$$

(29) $$\int H_{c}\left(A\left[\frac{\partial }{\partial t}m_{\theta }\left(y,t\right)-\tau \right]\right)H_{c}{\left(A\left[\frac{\partial }{\partial t}m_{\theta }\left(y,t\right)-\tau \right]\right)}^{⊤}dP_{0}\left(y\right)=I_{\ell +1}$$

where

(30) $$H_{c}\left(y\right):=\left\{\begin{array}{c} y\cdot \min\left(1,\frac{c}{∥y∥}\right)\;\mathrm{if}\;y\neq 0\\ 0\;\mathrm{if}\;y=0 \end{array}\right.$$

is the multidimensional Huber function, with c>0, $I_{\ell +1}$ the identity matrix, *A* is a (l+1)×(l+1) matrix, and $\tau \in R^{l+1}$. For fixed θ, this system admits a unique solution $\left(t,A,\tau \right)=(t_{\theta }\left(P_{0}\right),A_{\theta }\left(P_{0}\right),\tau_{\theta }\left(P_{0}\right))$ (according to [18], p. 17).

The multidimensional Huber function is useful to define robust estimators; it transforms each point outside a hypersphere of *c* radius to the nearest point of it and leaves those inside unchanged (see [26], p. 239, [27]). By applying the multidimensional Huber function to the function $y\mapsto \frac{\partial }{\partial t}m_{\theta }\left(y,t\right)$, together with considering the scale matrix $A_{\theta }$ and the shift vector $\tau_{\theta }$, a modification is produced there, where the norm exceeds the bound *c*, and in the meantime, the original $t_{\theta }$ remains the solution of the equation based on the new truncated function. For parametric models, the multidimensional Huber function was also used in other contexts, for example to define optimal B${}_{s}$-robust estimators or optimal B${}_{i}$-robust estimators (see [26], p. 244).

The above arguments can be used for each probability measure *P* from the moment condition model $M^{1}$. This context allows defining the truncated version of the function $y\mapsto \frac{\partial }{\partial t}m_{\theta }\left(y,t\right)$, which we denote by $\psi_{\theta }\left(y,t\right)$, such that the original $t_{\theta }\left(P_{0}\right)$, the solution of Equation (26), is also the solution of the equation $\int \psi_{\theta }\left(y,t_{\theta }\left(P_{0}\right)\right)dP_{0}\left(y\right)=0$.

For θ fixed and *P* a probability measure, the equation $\int \frac{\partial }{\partial t}m_{\theta }\left(y,t\right)dP\left(y\right)=0$ has a unique solution $t=t_{\theta }\left(P\right)\in \Lambda_{\theta }\left(P\right)$ assuring the supremum in the dual form of the divergence $D_{\varphi }\left(M_{\theta },P\right)$ (see [12]). For each *t*, we define the $A_{\theta }\left(t\right)$ and $\tau_{\theta }\left(t\right)$ solutions of the system:

(31) $$\;\int H_{c}\left(A_{\theta }\left(t\right)\left[\frac{\partial }{\partial t}m_{\theta }\left(y,t\right)-\tau_{\theta }\left(t\right)\right]\right)dP\left(y\right)=0$$

(32) $$\int H_{c}\left(A_{\theta }\left(t\right)\left[\frac{\partial }{\partial t}m_{\theta }\left(y,t\right)-\tau_{\theta }\left(t\right)\right]\right)H_{c}{\left(A_{\theta }\left(t\right)\left[\frac{\partial }{\partial t}m_{\theta }\left(y,t\right)-\tau_{\theta }\left(t\right)\right]\right)}^{⊤}dP\left(y\right)=I_{\ell +1}.$$

We define a new estimator ${\hat{t}}_{\theta }^{c}$ of $t_{\theta }=t_{\theta }\left(P_{0}\right)$, as a Z-estimator corresponding to the ψ-function:

(33) $$\psi_{\theta }\left(x,t\right):=H_{c}\left(A_{\theta }\left(t\right)\left[\frac{\partial }{\partial t}m_{\theta }\left(x,t\right)-\tau_{\theta }\left(t\right)\right]\right);$$

more precisely, ${\hat{t}}_{\theta }^{c}$ is defined by

(34) $$\int \psi_{\theta }\left(y,{\hat{t}}_{\theta }^{c}\right)dP_{n}\left(y\right)=0\;\;\mathrm{or}\;\;\sum_{i=1}^{n}H_{c}\left(A_{\theta }\left({\hat{t}}_{\theta }^{c}\right)\left[\frac{\partial }{\partial t}m_{\theta }\left(X_{i},{\hat{t}}_{\theta }^{c}\right)-\tau_{\theta }\left({\hat{t}}_{\theta }^{c}\right)\right]\right)=0,$$

the theoretical counterpart of this estimating equation being

(35) $$\int \psi_{\theta }\left(y,t_{\theta }\left(P_{0}\right)\right)dP_{0}\left(y\right)=0.$$

For a given probability measure *P*, the statistical functional $t_{\theta }^{c}\left(P\right)$ associated with the estimator ${\hat{t}}_{\theta }^{c}$, whenever it exists, is defined by

(36) $$\int \psi_{\theta }\left(y,t_{\theta }^{c}\left(P\right)\right)dP\left(y\right)=\int H_{c}\left(A_{\theta }\left(t_{\theta }^{c}\left(P\right)\right)\left[\frac{\partial }{\partial t}m_{\theta }\left(y,t_{\theta }^{c}\left(P\right)\right)-\tau_{\theta }\left(t_{\theta }^{c}\left(P\right)\right)\right]\right)dP\left(y\right)=0.$$

Note that

(37) $$t_{\theta }^{c}\left(P_{0}\right)=t_{\theta }\left(P_{0}\right),$$

by construction.

**Remark** **1.** *We notice a similarity between the Z-estimator defined in (34) and the classical optimal Bs-robust estimator for parametric models from [26]. In the case of the parametric models, the M-estimator corresponding to the ψ-function (33), but defined for the classical score function $\frac{\partial }{\partial t}\left(\ln f_{t}\left(x\right)\right)=\frac{\frac{\partial }{\partial t}f_{t}\left(x\right)}{f_{t}\left(x\right)}$ instead of the function $\frac{\partial }{\partial t}m_{\theta }\left(x,t\right)$ (inclusively in the system (31) and (32) defining $A_{\theta }\left(t\right)$ and $\tau_{\theta }\left(t\right)$), is the classical optimal Bs-robust estimator ($f_{t}\left(x\right)$ denotes the density corresponding to a parametric model indexed by the parameter t). The classical optimal Bs-robust estimator for parametric models has the optimal property that minimizes a measure of the asymptotic mean-squared error, among all the Fisher-consistent estimators with a self-standardized sensitivity smaller than the positive constant c.*

In the following, for a given divergence, using the estimators ${\hat{t}}_{\theta }^{c}$ for $t_{\theta }\left(P_{0}\right)$, we constructed new estimators of the parameter $\theta_{0}$ of the model. In Section 3.3, we prove that all the estimators ${\hat{t}}_{\theta }^{c}$ are robust, and this property will be transferred to the new estimators that we define for the parameter $\theta_{0}$.

To define new estimators for $\theta_{0}$, we used the dual representation (15) of the divergence $D_{\varphi }\left(M_{\theta },P_{0}\right)$. Since

(38) $$\begin{array}{ccc} \theta_{0}=\arg\underset{\theta \in \Theta }{\inf}D_{\varphi }\left(M_{\theta },P_{0}\right) & = & \arg\underset{\theta \in \Theta }{\inf}\underset{t\in \Lambda_{\theta }}{\sup}\int m_{\theta }\left(y,t\right)dP_{0}\left(y\right) \end{array}$$

(39) $$\begin{array}{ccc} & = & \arg\underset{\theta \in \Theta }{\inf}\int m_{\theta }\left(y,t_{\theta }\left(P_{0}\right)\right)dP_{0}\left(y\right), \end{array}$$

$\theta =\theta_{0}$ is the solution of the equation:

(40) $$\int \frac{\partial }{\partial \theta }\left[m\left(y,\theta ,t\left(\theta ,P_{0}\right)\right)\right]dP_{0}\left(y\right)=0,$$

where we used the notation $t\left(\theta ,P\right):=t_{\theta }\left(P\right)$. Equation (40) may be written as

(41) $$\int \frac{\partial }{\partial \theta }m\left(y,\theta_{0},t\left(\theta_{0},P_{0}\right)\right)dP_{0}\left(y\right)+\frac{\partial }{\partial \theta }t{\left(\theta_{0},P_{0}\right)}^{⊤}\int \frac{\partial }{\partial t}m\left(y,\theta_{0},t\left(\theta_{0},P_{0}\right)\right)dP_{0}\left(y\right)=0.$$

On the basis of the definition of $t_{\theta }\left(P_{0}\right)=t\left(\theta ,P_{0}\right)$, for $\theta =\theta_{0}$, we have

(42) $$\int \frac{\partial }{\partial t}m\left(y,\theta_{0},t\left(\theta_{0},P_{0}\right)\right)dP_{0}\left(y\right)=0;$$

therefore, we deduce that $\theta =\theta_{0}$ is the solution of equation:

(43) $$\int \frac{\partial }{\partial \theta }m\left(y,\theta ,t\left(\theta ,P_{0}\right)\right)dP_{0}\left(y\right)=0.$$

Using (37), namely $t^{c}\left(\theta ,P_{0}\right)=t\left(\theta ,P_{0}\right)$, we obtain that $\theta =\theta_{0}$ is in fact the solution of equation:

(44) $$\int \frac{\partial }{\partial \theta }m\left(y,\theta ,t^{c}\left(\theta ,P_{0}\right)\right)dP_{0}\left(y\right)=0.$$

Then, we define a new estimator ${\hat{\theta }}_{\varphi }^{c}$ of $\theta_{0}$, as a plug-in estimator solution of the equation:

(45) $$\int \frac{\partial }{\partial \theta }m\left(y,{\hat{\theta }}_{\varphi }^{c},t^{c}\left({\hat{\theta }}_{\varphi }^{c},P_{n}\right)\right)dP_{n}\left(y\right)=0.$$

For a probability measure *P*, the statistical functional $T^{c}$ corresponding to the estimator ${\hat{\theta }}_{\varphi }^{c}$, whenever it exists, is defined by

(46) $$\int \frac{\partial }{\partial \theta }m\left(y,T^{c}\left(P\right),t^{c}\left(T^{c}\left(P\right),P\right)\right)dP\left(y\right)=0.$$

The functional $T^{c}$ is Fisher-consistent, because

(47) $$T^{c}\left(P_{0}\right)=\theta_{0}.$$

This equality is obtained by using (46) for $P=P_{0}$, the fact that $t^{c}\left(T^{c}\left(P_{0}\right),P_{0}\right)=t\left(T^{c}\left(P_{0}\right),P_{0}\right)$, and the definition of $t_{\theta }\left(P_{0}\right)=t\left(\theta ,P_{0}\right)$ for $\theta =T^{c}\left(P_{0}\right)$, all these leading to

(48) $$\int \frac{\partial }{\partial \theta }m\left(y,T^{c}\left(P_{0}\right),t\left(T^{c}\left(P_{0}\right),P_{0}\right)\right)dP_{0}\left(y\right)+\frac{\partial }{\partial \theta }t{\left(T^{c}\left(P_{0}\right),P_{0}\right)}^{⊤}\int \frac{\partial }{\partial t}m\left(y,T^{c}\left(P_{0}\right),t\left(T^{c}\left(P_{0}\right),P_{0}\right)\right)dP_{0}\left(y\right)=0.$$

Since $\theta_{0}$ is the unique solution of Equation (41) and, according to (48), $T^{c}\left(P_{0}\right)$ would be another solution to the same equation, we deduce (47).

From (34) and (45), we have

$$\begin{array}{c} \int \psi_{{\hat{\theta }}_{\varphi }^{c}}\left(y,t^{c}\left({\hat{\theta }}_{\varphi }^{c},P_{n}\right)\right)dP_{n}\left(y\right)=0,\\ \int \frac{\partial }{\partial \theta }m\left(y,{\hat{\theta }}_{\varphi }^{c},t^{c}\left({\hat{\theta }}_{\varphi }^{c},P_{n}\right)\right)dP_{n}\left(y\right)=0, \end{array}$$

and then,

$$\begin{array}{c} \int \psi \left(y,{\hat{\theta }}_{\varphi }^{c},{\hat{t}}_{{\hat{\theta }}_{\varphi }^{c}}^{c}\right)dP_{n}\left(y\right)=0,\\ \int \frac{\partial }{\partial \theta }m\left(y,{\hat{\theta }}_{\varphi }^{c},{\hat{t}}_{{\hat{\theta }}_{\varphi }^{c}}^{c}\right)dP_{n}\left(y\right)=0, \end{array}$$

with $\psi \left(y,\theta ,t\right):=\psi_{\theta }\left(y,t\right)$. The couple of estimators ${\hat{\theta }}_{\varphi }^{c},{\hat{t}}_{{\hat{\theta }}_{\varphi }^{c}}^{c}$ can be viewed as a Z-estimator solution of the above system. Denoting

(49) $$\Psi \left(y,\theta ,t\right):={\left(\psi {\left(y,\theta ,t\right)}^{⊤},{\left(\frac{\partial }{\partial \theta }m\left(y,\theta ,t\right)\right)}^{⊤}\right)}^{⊤},$$

the Z-estimators ${\hat{\theta }}_{\varphi }^{c},{\hat{t}}_{{\hat{\theta }}_{\varphi }^{c}}^{c}$ are the solutions of the system:

(50) $$\int \Psi \left(y,{\hat{\theta }}_{\varphi }^{c},{\hat{t}}_{{\hat{\theta }}_{\varphi }^{c}}^{c}\right)dP_{n}\left(y\right)=0,$$

and the theoretical counterpart is given by

(51) $$\int \Psi \left(y,\theta_{0},t_{\theta_{0}}\right)dP_{0}\left(y\right)=0.$$

### 3.2. Asymptotic Properties

In this section, we establish the consistency and the asymptotic distributions for the estimators ${\hat{\theta }}_{\varphi }^{c}$ and ${\hat{t}}_{{\hat{\theta }}_{\varphi }^{c}}^{c}$. In order to prove the consistency of the estimators, we adopted the results from the general theory of Z-estimators as presented for example in [28]. Then, using the consistency of the estimators, as well as supplementary conditions, we proved that the asymptotic distributions of the estimators are multivariate normal:

**Assumption A1.**

*(a)*  *There exist compact neighbourhoods $V_{\theta_{0}}$ of $\theta_{0}$ and $V_{t_{\theta_{0}}}$ of $t_{\theta_{0}}$ such that*
$$\int \underset{\theta \in V_{\theta_{0}},t\in V_{t_{\theta_{0}}}}{\sup}∥\Psi \left(y,\theta ,t\right)∥dP_{0}\left(y\right)<\infty .$$
*(b)*  *For any positive ε, the following condition holds*
$$\underset{(\theta ,t)\in M}{\inf}∥\int \Psi \left(y,\theta ,t\right)∥dP_{0}\left(y\right)>0=∥\int \Psi \left(y,\theta_{0},t_{\theta_{0}}\right)dP_{0}\left(y\right)∥,$$
*where $M:=\{\left(\theta ,t\right)\;s.t.\;∥\left(\theta ,t\right)-\left(\theta_{0},t_{\theta_{0}}\right)∥>\varepsilon \}$.*

**Proposition** **1.** *Under Assumption 1, ${\hat{\theta }}_{\varphi }^{c}$ converges in probability to $\theta_{0}$ and ${\hat{t}}_{{\hat{\theta }}_{\varphi }^{c}}^{c}$ converges in probability to $t_{\theta_{0}}$:*

**Assumption 2.**

*(a)*  *Both estimators ${\hat{\theta }}_{\varphi }^{c}$ and ${\hat{t}}_{{\hat{\theta }}_{\varphi }^{c}}^{c}$ converge in probability to $\theta_{0}$ and $t_{\theta_{0}}$, respectively.*
*(b)*  *The function (θ,t)↦ψ(x,θ,t) is $C^{2}$ on some neighbourhood $V(\theta_{0},t_{\theta_{0}})$ for all x ($P_{0}$ a.s.), and the partial derivatives of order two of the functions $\left\{\left(\theta ,t\right)\mapsto \psi \left(x,\theta ,t\right);\left(\theta ,t\right)\in V\left(\theta_{0},t_{\theta_{0}}\right)\right\}$ are dominated by some $P_{0}$-integrable function $H_{1}\left(x\right)$.*
*(c)*  *The function (θ,t)↦m(x,θ,t) is $C^{3}$ on some neighbourhood $U(\theta_{0},t_{\theta_{0}})$ for all x ($P_{0}$ a.s.), and the partial derivatives of order three of the functions $\left\{\left(\theta ,t\right)\mapsto m\left(x,\theta ,t\right);\left(\theta ,t\right)\in U\left(\theta_{0},t_{\theta_{0}}\right)\right\}$ are dominated by some $P_{0}$-integrable function $H_{2}\left(x\right)$.*
*(d)*  *$\int ∥\frac{\partial }{\partial \theta }m\left(y,\theta_{0},t_{\theta_{0}}\right){∥}^{2}dP_{0}\left(y\right)$ is finite, and the matrix:*
(52) $$S:=\left(\begin{array}{cc} S_{11} & S_{12}\\ S_{21} & S_{22} \end{array}\right),$$
*with $S_{11}:={\left(\int \frac{\partial }{\partial t}\psi \left(y,\theta_{0},t_{\theta_{0}}\right)dP_{0}\left(y\right)\right)}^{⊤}$, $S_{12}:={\left(\int \frac{\partial }{\partial \theta }\psi \left(y,\theta_{0},t_{\theta_{0}}\right)dP_{0}\left(y\right)\right)}^{⊤}$, $S_{21}:={\left(\int \frac{\partial^{2}}{\partial \theta \partial t}m\left(y,\theta_{0},t_{\theta_{0}}\right)dP_{0}\left(y\right)\right)}^{⊤}$ and $S_{22}:=\int \frac{\partial^{2}}{\partial^{2}\theta }m\left(y,\theta_{0},t_{\theta_{0}}\right)dP_{0}\left(y\right)$, exists and is invertible.*

**Proposition** **2.** *Let $P_{0}$ belong to the model $M^{1}$, and suppose that Assumption 2 holds. Then, both $\sqrt{n}\left({\hat{\theta }}_{\varphi }^{c}-\theta_{0}\right)$ and $\sqrt{n}\left({\hat{t}}_{{\hat{\theta }}_{\varphi }^{c}}^{c}-t_{\theta_{0}}\right)$ converge in distribution to a centred multivariate normal variable with covariance matrices given by*

(53) $$\left[{\left[S_{21}S_{11}^{-1}S_{12}\right]}^{-1}S_{21}S_{11}^{-1}\right]\times {\left[{\left[S_{21}S_{11}^{-1}S_{12}\right]}^{-1}S_{21}S_{11}^{-1}\right]}^{⊤},$$

*and*

(54) $$\left[S_{11}^{-1}-S_{11}^{-1}S_{12}{\left[S_{21}S_{11}^{-1}S_{12}\right]}^{-1}S_{21}S_{11}^{-1}\right]\times {\left[S_{11}^{-1}-S_{11}^{-1}S_{12}{\left[S_{21}S_{11}^{-1}S_{12}\right]}^{-1}S_{21}S_{11}^{-1}\right]}^{⊤}.$$

The condition of Type (a) from Assumption 1 is usually considered to apply the uniform law of large numbers. For many choices of divergence (for example, those from the Cressie–Read family), the function Ψ is continuous in (θ,t), and consequently, this condition is verified. The second condition from Assumption 1 is imposed for the uniqueness of $\left(\theta_{0},t_{\theta_{0}}\right)$ as a solution of the equation and is verified, for example, whenever Ψ is continuous and the parameter space is compact ([28], p. 46). Furthermore, the conditions of Type (b)–(d), included in Assumption 2, are often imposed in order to apply the law of large numbers or the central limit theorem and can be verified for the functions appearing in the definitions of estimators proposed in the present paper.

### 3.3. Influence Functions and Robustness

In this section, we derive the influence functions of the estimators ${\hat{t}}_{\theta }^{c}$ and ${\hat{\theta }}_{\varphi }^{c}$ and prove their B-robustness. The corresponding statistical functionals are defined by (36) and (46), respectively.

Recall that, a map *T*, defined on a set of probability measures and parameter-space-valued, is a statistical functional corresponding to an estimator $\hat{\theta }$ of the parameter $\theta_{0}$ from the model $P_{0}$, if $\hat{\theta }=T\left(P_{n}\right)$, $P_{n}$ being the empirical measure corresponding to the sample. The influence function of *T* at $P_{0}$ is defined by

$$\mathrm{IF}\left(x;T,P_{0}\right):={\left.\frac{\partial T({\tilde{P}}_{\varepsilon x})}{\partial \varepsilon }\right|}_{\varepsilon =0},$$

where ${\tilde{P}}_{\varepsilon x}:=\left(1-\varepsilon \right)\;P_{0}+\varepsilon \;\delta_{x}$, $\delta_{x}$ being the Dirac measure. An unbounded influence function implies an unbounded asymptotic bias of a statistic under single-point contamination of the model. Therefore, a natural robustness requirement on a statistical functional is the boundedness of its influence function. Whenever the influence function is bounded with respect to *x*, the corresponding estimator is called B-robust [26].

**Proposition** **3.** *For fixed θ, the influence function of the functional $t_{\theta }^{c}$ is given by*

(55) $$\mathrm{IF}\left(x;t_{\theta }^{c},P_{0}\right)=-{\left\{\int \frac{\partial }{\partial t}\psi_{\theta }\left(y,t_{\theta }\left(P_{0}\right)\right)dP_{0}\left(y\right)\right\}}^{-1}\cdot \psi_{\theta }\left(x,t_{\theta }\left(P_{0}\right)\right).$$

**Proposition** **4.** *The influence function of the functional $T^{c}$ is given by*

(56) $$\begin{array}{c} \mathrm{IF}\left(x;T^{c},P_{0}\right)\;\;=\;\;{\left\{{\left[\frac{\partial }{\partial \theta }\bar{g}\left(y,\theta_{0}\right)dP_{0}\left(y\right)\right]}^{⊤}{\left[\int \bar{g}\left(y,\theta_{0}\right)\bar{g}{\left(y,\theta_{0}\right)}^{⊤}dP_{0}\left(y\right)\right]}^{-1}\left[\frac{\partial }{\partial \theta }\bar{g}\left(y,\theta_{0}\right)dP_{0}\left(y\right)\right]\right\}}^{-1}\\ \;\;\cdot {\left[\frac{\partial }{\partial \theta }\bar{g}\left(y,\theta_{0}\right)dP_{0}\left(y\right)\right]}^{⊤}\frac{1}{\varphi^{\prime \prime }\left(1\right)}\mathrm{IF}\left(x;t_{\theta_{0}}^{c},P_{0}\right). \end{array}$$

On the basis of Propositions 3 and 4, since $x\mapsto \psi_{\theta }\left(x,t_{\theta }\left(P_{0}\right)\right)$ is bounded, all the estimators ${\hat{\theta }}_{\varphi }^{c}$ are B-robust.

## 4. Conclusions

We introduced a class of robust Z-estimators for moment condition models. These new estimators can be seen as robust alternatives for the minimum empirical divergence estimators. By using truncated functions based on the multidimensional Huber function, we defined robust estimators of the element that realizes the supremum in the dual form of the divergence, as well as new robust estimators for the parameter of the model. The asymptotic properties were proven, including the consistency and the limit laws. The influence functions for all the proposed estimators are bounded; therefore, these estimators are B-robust. The truncated function that we used to define the new robust Z-estimators contains functions implicitly defined, for which analytic forms are not available. The implementation of the estimation method will be addressed in a future research study. The idea of using the multidimensional Huber function, together with a scale matrix and a shift vector, to create a bounded version of the function corresponding to the estimating equation for the parameter of interest, could be considered in other contexts as well and would lead to new robust Z-estimators. As one of the Referees suggested, some other bounded functions could be used to define new robust Z-estimators for moment condition models. For example, the Tukey biweight function used together with a norm inside, in order to be appropriate to be applied to functions with vector values, could also be considered. Again, the original parameter of interest should remain the solution of the estimating equation based on the new bounded function. Such an idea is interesting to be analysed in future studies, in order to provide new robust versions of minimum empirical divergence estimators or robust Z-estimators in other contexts.

## Data Availability

Not applicable.

## Abbreviations

The following abbreviations are used in this manuscript:

i.i.d.	independent and identically distributed
a.c.	absolutely continuous
GMM	generalized method of moments
CU	continuous updating
EL	empirical likelihood
ET	exponential tilting
GEL	generalized empirical likelihood
ETEL	exponentially tilted empirical likelihood

## Appendix A

**Proof** **of** **Proposition** **1.** Since (θ,t)↦Ψ(y,θ,t) is continuous, by the uniform law of large numbers, Assumption 1 (a) implies

(A1) $$\underset{\theta \in V_{\theta_{0},t\in V_{t_{\theta_{0}}}}}{\sup}∥\int \Psi \left(y,\theta ,t\right)dP_{n}\left(y\right)-\int \Psi \left(y,\theta ,t\right)dP_{0}\left(y\right)∥\to 0,$$

in probability. This result together with Assumption 1 (b) ensures the convergence in probability of the estimators ${\hat{\theta }}_{\varphi }^{c}$ and ${\hat{t}}_{{\hat{\theta }}_{\varphi }^{c}}^{c}$ toward $\theta_{0}$ and $t_{\theta_{0}}$, respectively. The proof is the same as the one for Theorem 5.9 from [28], p. 46. □

**Proof** **of** **Proposition** **2.** By the definitions of ${\hat{\theta }}_{\varphi }^{c}$ and ${\hat{t}}_{{\hat{\theta }}_{\varphi }^{c}}^{c}$, they both satisfy

$$\int \psi \left(y,{\hat{\theta }}_{\varphi }^{c},{\hat{t}}_{{\hat{\theta }}_{\varphi }^{c}}^{c}\right)dP_{n}\left(y\right)=0\;\;\;\left(E1\right)$$

$$\int \frac{\partial }{\partial \theta }m\left(y,{\hat{\theta }}_{\varphi }^{c},{\hat{t}}_{{\hat{\theta }}_{\varphi }^{c}}^{c}\right)dP_{n}\left(y\right)=0\;\;\;\left(E2\right)$$

Using a Taylor expansion in (*E*1), there exists $\left({\tilde{\theta }}_{\varphi }^{c},{\tilde{t}}_{\varphi }^{c}\right)$ inside the segment that links $\left({\hat{\theta }}_{\varphi }^{c},{\hat{t}}_{{\hat{\theta }}_{\varphi }^{c}}^{c}\right)$ and $\left(\theta_{0},t_{\theta_{0}}\right)$ such that

(A2) $$\begin{array}{c} 0=\int \psi \left(y,\theta_{0},t_{\theta_{0}}\right)dP_{n}\left(y\right)+\left[{\left(\int \frac{\partial }{\partial t}\psi \left(y,\theta_{0},t_{\theta_{0}}\right)dP_{n}\left(y\right)\right)}^{⊤},\right.\\ \;\left.{\left(\int \frac{\partial }{\partial \theta }\psi \left(y,\theta_{0},t_{\theta_{0}}\right)dP_{n}\left(y\right)\right)}^{⊤}\right]\cdot a_{n}+\frac{1}{2}a_{n}^{⊤}A_{n}a_{n}, \end{array}$$

where

(A3) $$a_{n}:={\left({\left({\hat{t}}_{{\hat{\theta }}_{\varphi }^{c}}^{c}-t_{\theta_{0}}\right)}^{⊤},{\left({\hat{\theta }}_{\varphi }^{c}-\theta_{0}\right)}^{⊤}\right)}^{⊤},$$

and

(A4) $$A_{n}:=\left(\begin{array}{cc} \int \frac{\partial^{2}}{\partial^{2}t}\psi \left(y,{\tilde{\theta }}_{\varphi }^{c},{\tilde{t}}_{\varphi }^{c}\right)dP_{n}\left(y\right) & \int \frac{\partial^{2}}{\partial \theta \partial t}\psi \left(y,{\tilde{\theta }}_{\varphi }^{c},{\tilde{t}}_{\varphi }^{c}\right)dP_{n}\left(y\right)\\ \int \frac{\partial^{2}}{\partial t\partial \theta }\psi \left(y,{\tilde{\theta }}_{\varphi }^{c},{\tilde{t}}_{\varphi }^{c}\right)dP_{n}\left(y\right) & \int \frac{\partial^{2}}{\partial^{2}\theta }\psi \left(y,{\tilde{\theta }}_{\varphi }^{c},{\tilde{t}}_{\varphi }^{c}\right)dP_{n}\left(y\right) \end{array}\right).$$

By Assumption 2 (b), the law of large numbers implies that $A_{n}=O_{P}\left(1\right)$. Then, using Assumption 2 (a), the last term in (A2) can be written $o_{P}\left(1\right)a_{n}.$ On the other hand, by Assumption 2 (d), using the law of large numbers, we can write

$$\begin{array}{c} \left[{\left(\int \frac{\partial }{\partial t}\psi \left(y,\theta_{0},t_{\theta_{0}}\right)dP_{n}\left(y\right)\right)}^{⊤},{\left(\int \frac{\partial }{\partial \theta }\psi \left(y,\theta_{0},t_{\theta_{0}}\right)dP_{n}\left(y\right)\right)}^{⊤}\right]\\ =\left[{\left(\int \frac{\partial }{\partial t}\psi \left(y,\theta_{0},t_{\theta_{0}}\right)dP_{0}\left(y\right)\right)}^{⊤},{\left(\int \frac{\partial }{\partial \theta }\psi \left(y,\theta_{0},t_{\theta_{0}}\right)dP_{0}\left(y\right)\right)}^{⊤}\right]+o_{P}\left(1\right). \end{array}$$

Consequently, (A2) becomes

(A5) $$\begin{array}{c} -\int \psi \left(y,\theta_{0},t_{\theta_{0}}\right)dP_{n}\left(y\right)\\ =\left[{\left(\int \frac{\partial }{\partial t}\psi \left(y,\theta_{0},t_{\theta_{0}}\right)dP_{0}\left(y\right)\right)}^{⊤}+o_{P}\left(1\right),{\left(\int \frac{\partial }{\partial \theta }\psi \left(y,\theta_{0},t_{\theta_{0}}\right)dP_{0}\left(y\right)\right)}^{⊤}+o_{P}\left(1\right)\right]\cdot a_{n}. \end{array}$$

In the same way, using a Taylor expansion in (E2), there exists $\left({\bar{\theta }}_{\varphi }^{c},{\bar{t}}_{\varphi }^{c}\right)$ inside the segment that links $\left({\hat{\theta }}_{\varphi }^{c},{\hat{t}}_{{\hat{\theta }}_{\varphi }^{c}}^{c}\right)$ and $\left(\theta_{0},t_{\theta_{0}}\right)$ such that

(A6) $$\begin{array}{c} 0=\int \frac{\partial }{\partial \theta }m\left(y,\theta_{0},t_{\theta_{0}}\right)dP_{n}\left(y\right)+\left[{\left(\int \frac{\partial^{2}}{\partial \theta \partial t}m\left(y,\theta_{0},t_{\theta_{0}}\right)dP_{n}\left(y\right)\right)}^{⊤},\right.\\ \;\left.{\left(\int \frac{\partial^{2}}{\partial^{2}\theta }m\left(y,\theta_{0},t_{\theta_{0}}\right)dP_{n}\left(y\right)\right)}^{⊤}\right]\cdot a_{n}+\frac{1}{2}a_{n}^{⊤}B_{n}a_{n}, \end{array}$$

where

(A7) $$B_{n}:=\left(\begin{array}{cc} \int \frac{\partial^{3}}{\partial \theta \partial^{2}t}m\left(y,{\bar{\theta }}_{\varphi }^{c},{\bar{t}}_{\varphi }^{c}\right)dP_{n}\left(y\right) & \int \frac{\partial^{3}}{\partial^{2}\theta \partial t}m\left(y,{\bar{\theta }}_{\varphi }^{c},{\bar{t}}_{\varphi }^{c}\right)dP_{n}\left(y\right)\\ \int \frac{\partial^{3}}{\partial \theta \partial t\partial \theta }m\left(y,{\bar{\theta }}_{\varphi }^{c},{\tilde{t}}_{\varphi }^{c}\right)dP_{n}\left(y\right) & \int \frac{\partial^{3}}{\partial^{3}\theta }m\left(y,{\bar{\theta }}_{\varphi }^{c},{\bar{t}}_{\varphi }^{c}\right)dP_{n}\left(y\right) \end{array}\right).$$

Similarly, as in (A5), we obtain

(A8) $$\begin{array}{c} -\int \frac{\partial }{\partial \theta }m\left(y,\theta_{0},t_{\theta_{0}}\right)dP_{n}\left(y\right)\\ =\left[{\left(\int \frac{\partial^{2}}{\partial \theta \partial t}m\left(y,\theta_{0},t_{\theta_{0}}\right)dP_{0}\left(y\right)\right)}^{⊤}+o_{P}\left(1\right),\int \frac{\partial^{2}}{\partial^{2}\theta }m\left(y,\theta_{0},t_{\theta_{0}}\right)dP_{0}\left(y\right)+o_{P}\left(1\right)\right]\cdot a_{n}. \end{array}$$

Using (A5) and (A8), we obtain

(A9) $$\begin{array}{c} \sqrt{n}a_{n}=\sqrt{n}{\left(\begin{array}{cc} {\left(\int \frac{\partial }{\partial t}\psi \left(y,\theta_{0},t_{\theta_{0}}\right)dP_{0}\left(y\right)\right)}^{⊤} & {\left(\int \frac{\partial }{\partial \theta }\psi \left(y,\theta_{0},t_{\theta_{0}}\right)dP_{0}\left(y\right)\right)}^{⊤}\\ {\left(\int \frac{\partial^{2}}{\partial \theta \partial t}m\left(y,\theta_{0},t_{\theta_{0}}\right)dP_{n}\left(y\right)\right)}^{⊤} & \int \frac{\partial^{2}}{\partial^{2}\theta }m\left(y,\theta_{0},t_{\theta_{0}}\right)dP_{n}\left(y\right) \end{array}\right)}^{-1}\\ \;\times \left(\begin{array}{c} -\int \psi \left(y,\theta_{0},t_{\theta_{0}}\right)dP_{n}\left(y\right)\\ -\int \frac{\partial }{\partial \theta }m\left(y,\theta_{0},t_{\theta_{0}}\right)dP_{n}\left(y\right) \end{array}\right)+o_{P}\left(1\right). \end{array}$$

Consider *S* the (l+1+d)×(l+1+d) matrix:

(A10) $$S:=\left(\begin{array}{cc} S_{11} & S_{12}\\ S_{21} & S_{22} \end{array}\right),$$

with $S_{11}:={\left(\int \frac{\partial }{\partial t}\psi \left(y,\theta_{0},t_{\theta_{0}}\right)dP_{0}\left(y\right)\right)}^{⊤}$, $S_{12}:={\left(\int \frac{\partial }{\partial \theta }\psi \left(y,\theta_{0},t_{\theta_{0}}\right)dP_{0}\left(y\right)\right)}^{⊤}$, $S_{21}:={\left(\int \frac{\partial^{2}}{\partial \theta \partial t}m\left(y,\theta_{0},t_{\theta_{0}}\right)dP_{0}\left(y\right)\right)}^{⊤}$, and $S_{22}:=\int \frac{\partial^{2}}{\partial^{2}\theta }m\left(y,\theta_{0},t_{\theta_{0}}\right)dP_{0}\left(y\right)$. Through calculations, we have

(A11) $$\begin{array}{ccc} S_{21} & = & -[0_{d},\int \frac{\partial }{\partial \theta }g\left(y,\theta_{0}\right)dP_{0}\left(y\right)], \end{array}$$

(A12) $$\begin{array}{ccc} S_{22} & = & [0_{d},\ldots ,0_{d}]. \end{array}$$

From (A9), we deduce that

(A13) $$\sqrt{n}\left(\begin{array}{c} {\hat{t}}_{{\hat{\theta }}_{\varphi }^{c}}^{c}-t_{\theta_{0}}\\ {\hat{\theta }}_{\varphi }^{c}-\theta_{0} \end{array}\right)=S^{-1}\sqrt{n}\left(\begin{array}{c} -\int \psi \left(y,\theta_{0},t_{\theta_{0}}\right)dP_{n}\left(y\right)\\ 0_{d} \end{array}\right)+o_{P}\left(1\right).$$

On the other hand, under assumption Assumption 2 (d), using the central limit theorem,

(A14) $$\sqrt{n}\left(\begin{array}{c} -\int \psi \left(y,\theta_{0},t_{\theta_{0}}\right)dP_{n}\left(y\right)\\ 0_{d} \end{array}\right)$$

converges in distribution to a centred multivariate normal variable with covariance matrix:

(A15) $$M:=\left(\begin{array}{cc} M_{11} & M_{12}\\ M_{21} & M_{22} \end{array}\right),$$

with

$$M_{11}:=\mathrm{cov}\left[\psi \left(X,\theta_{0},t_{\theta_{0}}\right)\right],\;\;M_{12}:=\left(\begin{array}{c} 0_{d}^{⊤}\\ \vdots \\ 0_{d}^{⊤} \end{array}\right),\;\;M_{21}:=\left(\begin{array}{c} 0\\ \vdots \\ 0 \end{array}\;\;\begin{array}{c} 0_{l}^{⊤}\\ \vdots \\ 0_{l}^{⊤} \end{array}\right),\;\;M_{22}:=\left(\begin{array}{c} 0_{d}^{⊤}\\ \vdots \\ 0_{d}^{⊤} \end{array}\right).$$

Since $E[\psi \left(X,\theta_{0},t_{\theta_{0}}\right)]=0$ by the construction of ψ, we obtain

(A16) $$M_{11}=\mathrm{cov}\left[\psi \left(X,\theta_{0},t_{\theta_{0}}\right)\right]=\int \psi \left(y,\theta_{0},t_{\theta_{0}}\right)\psi {\left(y,\theta_{0},t_{\theta_{0}}\right)}^{⊤}dP_{0}\left(y\right)=I_{l+1},$$

on the basis of (29) for $\theta =\theta_{0}$. Using then (A13) and the Slutsky theorem, we obtain that

(A17) $$\sqrt{n}\left(\begin{array}{c} {\hat{t}}_{{\hat{\theta }}_{\varphi }^{c}}^{c}-t_{\theta_{0}}\\ {\hat{\theta }}_{\varphi }^{c}-\theta_{0} \end{array}\right)$$

converges in distribution to a centred multivariate normal variable with the covariance matrix given by

(A18) $$C=S^{-1}M{\left[S^{-1}\right]}^{⊤}.$$

If we denote

(A19) $$C:=\left(\begin{array}{cc} C_{11} & C_{12}\\ C_{21} & C_{22} \end{array}\right),$$

through calculation, we obtain

(A20) $$\begin{array}{ccc} C_{11} & = & \left[S_{11}^{-1}-S_{11}^{-1}S_{12}{\left[S_{21}S_{11}^{-1}S_{12}\right]}^{-1}S_{21}S_{11}^{-1}\right]\times {\left[S_{11}^{-1}-S_{11}^{-1}S_{12}{\left[S_{21}S_{11}^{-1}S_{12}\right]}^{-1}S_{21}S_{11}^{-1}\right]}^{⊤}, \end{array}$$

(A21) $$\begin{array}{ccc} C_{12} & = & \left[S_{11}^{-1}-S_{11}^{-1}S_{12}{\left[S_{21}S_{11}^{-1}S_{12}\right]}^{-1}S_{21}S_{11}^{-1}\right]\times {\left[{\left[S_{21}S_{11}^{-1}S_{12}\right]}^{-1}S_{21}S_{11}^{-1}\right]}^{⊤}, \end{array}$$

(A22) $$\begin{array}{ccc} C_{21} & = & \left[{\left[S_{21}S_{11}^{-1}S_{12}\right]}^{-1}S_{21}S_{11}^{-1}\right]\times {\left[S_{11}^{-1}-S_{11}^{-1}S_{12}{\left[S_{21}S_{11}^{-1}S_{12}\right]}^{-1}S_{21}S_{11}^{-1}\right]}^{⊤}, \end{array}$$

(A23) $$\begin{array}{ccc} C_{22} & = & \left[{\left[S_{21}S_{11}^{-1}S_{12}\right]}^{-1}S_{21}S_{11}^{-1}\right]\times {\left[{\left[S_{21}S_{11}^{-1}S_{12}\right]}^{-1}S_{21}S_{11}^{-1}\right]}^{⊤}. \end{array}$$

□

**Proof** **of** **Proposition** **3.** For the contaminated model ${\tilde{P}}_{\varepsilon x}=\left(1-\varepsilon \right)P_{0}+\varepsilon \delta_{x}$, whenever it exists, $t_{\theta }^{c}\left({\tilde{P}}_{\varepsilon x}\right)$ is defined as the solution of equation:

(A24) $$\int H_{c}\left(A_{\theta }\left(t_{\theta }^{c}\left({\tilde{P}}_{\varepsilon x}\right)\right)\left[\frac{\partial }{\partial t}m_{\theta }\left(y,t_{\theta }^{c}\left({\tilde{P}}_{\varepsilon x}\right)\right)-\tau_{\theta }\left(t_{\theta }^{c}\left({\tilde{P}}_{\varepsilon x}\right)\right)\right]\right)d{\tilde{P}}_{\varepsilon x}\left(y\right)=0.$$

It follows that

(A25) $$\begin{array}{c} \left(1-\varepsilon \right)\int H_{c}\left(A_{\theta }\left(t_{\theta }^{c}\left({\tilde{P}}_{\varepsilon x}\right)\right)\left[\frac{\partial }{\partial t}m_{\theta }\left(y,t_{\theta }^{c}\left({\tilde{P}}_{\varepsilon x}\right)\right)-\tau_{\theta }\left(t_{\theta }^{c}\left({\tilde{P}}_{\varepsilon x}\right)\right)\right]\right)dP_{0}\left(y\right)\\ +\varepsilon H_{c}\left(A_{\theta }\left(t_{\theta }^{c}\left({\tilde{P}}_{\varepsilon x}\right)\right)\left[\frac{\partial }{\partial t}m_{\theta }\left(x,t_{\theta }^{c}\left({\tilde{P}}_{\varepsilon x}\right)\right)-\tau_{\theta }\left(t_{\theta }^{c}\left({\tilde{P}}_{\varepsilon x}\right)\right)\right]\right)=0. \end{array}$$

Derivation with respect to ε in (A25) yields

(A26) $$\begin{array}{c} -\int H_{c}\left(A_{\theta }\left(t_{\theta }\left(P_{0}\right)\right)\left[\frac{\partial }{\partial t}m_{\theta }\left(y,t_{\theta }\left(P_{0}\right)\right)-\tau_{\theta }\left(t_{\theta }\left(P_{0}\right)\right)\right]\right)dP_{0}\left(y\right)\\ +\int \frac{\partial }{\partial t}{\left[H_{c}\left(A_{\theta }\left(t\right)\left[\frac{\partial }{\partial t}m_{\theta }\left(y,t\right)-\tau_{\theta }\left(t\right)\right]\right)\right]}_{t=t_{\theta }\left(P_{0}\right)}dP_{0}\left(y\right)\mathrm{IF}\left(x;t_{\theta }^{c},P_{0}\right)\\ +H_{c}\left(A_{\theta }\left(t_{\theta }\left(P_{0}\right)\right)\left[\frac{\partial }{\partial t}m_{\theta }\left(x,t_{\theta }\left(P_{0}\right)\right)-\tau_{\theta }\left(t_{\theta }\left(P_{0}\right)\right)\right]\right). \end{array}$$

Since the first integral in (A26) equals zero, we obtain

(A27) $$\begin{array}{c} \;\mathrm{IF}\left(x;t_{\theta }^{c},P_{0}\right)=-{\left\{\int \frac{\partial }{\partial t}{\left[H_{c}\left(A_{\theta }\left(t\right)\left[\frac{\partial }{\partial t}m_{\theta }\left(y,t\right)-\tau_{\theta }\left(t\right)\right]\right)\right]}_{t=t_{\theta }\left(P_{0}\right)}dP_{0}\left(y\right)\right\}}^{-1}\\ \;\cdot H_{c}\left(A_{\theta }\left(t_{\theta }\left(P_{0}\right)\right)\left[\frac{\partial }{\partial t}m_{\theta }\left(x,t_{\theta }\left(P_{0}\right)\right)-\tau_{\theta }\left(t_{\theta }\left(P_{0}\right)\right)\right]\right)\\ \;=-{\left\{\int \frac{\partial }{\partial t}\psi_{\theta }\left(y,t_{\theta }\left(P_{0}\right)\right)dP_{0}\left(y\right)\right\}}^{-1}\cdot \psi_{\theta }\left(x,t_{\theta }\left(P_{0}\right)\right). \end{array}$$

For each θ, the influence function (55) is bounded with respect to *x*; therefore, the estimators ${\hat{t}}_{\theta }^{c}$ are B-robust. □

**Proof** **of** **Proposition** **4.** For the contaminated model ${\tilde{P}}_{\varepsilon x}=\left(1-\varepsilon \right)P_{0}+\varepsilon \delta_{x}$, $T^{c}\left({\tilde{P}}_{\varepsilon x}\right)$ is defined as the solution of equation:

(A28) $$\int \frac{\partial }{\partial \theta }m\left(y,T^{c}\left({\tilde{P}}_{\varepsilon x}\right),t^{c}\left(T^{c}\left({\tilde{P}}_{\varepsilon x}\right),{\tilde{P}}_{\varepsilon x}\right)\right)d{\tilde{P}}_{\varepsilon x}\left(y\right)=0,$$

whenever this solution exists. Then,

(A29) $$\left(1-\varepsilon \right)\int \frac{\partial }{\partial \theta }m\left(y,T^{c}\left({\tilde{P}}_{\varepsilon x}\right),t^{c}\left(T^{c}\left({\tilde{P}}_{\varepsilon x}\right),{\tilde{P}}_{\varepsilon x}\right)\right)dP_{0}\left(y\right)+\varepsilon \frac{\partial }{\partial \theta }m\left(x,T^{c}\left({\tilde{P}}_{\varepsilon x}\right),t^{c}\left(T^{c}\left({\tilde{P}}_{\varepsilon x}\right),{\tilde{P}}_{\varepsilon x}\right)\right)=0.$$

Derivation with respect to ε in (A29) yields

(A30) $$\begin{array}{c} -\int \frac{\partial }{\partial \theta }m\left(y,\theta_{0},t^{c}\left(\theta_{0},P_{0}\right)\right)dP_{0}\left(y\right)+\int \frac{\partial^{2}}{\partial^{2}\theta }m{\left[y,\theta ,t\right]}_{\theta =\theta_{0},t=t_{\theta_{0}}\left(P_{0}\right)}dP_{0}\left(y\right)\mathrm{IF}\left(x;T^{c},P_{0}\right)+\\ +\int \frac{\partial^{2}}{\partial \theta \partial t}m{\left[y,\theta ,t\right]}_{\theta =\theta_{0},t=t_{\theta_{0}}\left(P_{0}\right)}dP_{0}\left(y\right)\cdot \left\{\frac{\partial }{\partial \theta }t^{c}\left(\theta_{0},P_{0}\right)\mathrm{IF}\left(x;T^{c},P_{0}\right)+\right.\\ \left.+\mathrm{IF}(x;t_{\theta_{0}}^{c},P_{0})\right\}+\frac{\partial }{\partial \theta }{\left[m\left(x,\theta ,t\right)\right]}_{\theta =\theta_{0},t=t_{\theta_{0}}\left(P_{0}\right)}=0. \end{array}$$

Some calculations show that

(A31) $$\frac{\partial }{\partial \theta }{\left[m\left(x,\theta ,t\right)\right]}_{\theta =\theta_{0},t=t_{\theta_{0}}\left(P_{0}\right)}=0\;\;\mathrm{and}\;\;\frac{\partial^{2}}{\partial^{2}\theta }{\left[m\left(x,\theta ,t\right)\right]}_{\theta =\theta_{0},t=t_{\theta_{0}}\left(P_{0}\right)}=0,$$

for any *x*; therefore, (A30) reduces to

(A32) $$\int \frac{\partial^{2}}{\partial \theta \partial t}m{\left[y,\theta ,t\right]}_{\theta =\theta_{0},t=t_{\theta_{0}}\left(P_{0}\right)}dP_{0}\left(y\right)\cdot \left\{\frac{\partial }{\partial \theta }t^{c}\left(\theta_{0},P_{0}\right)\mathrm{IF}\left(x;T^{c},P_{0}\right)+\mathrm{IF}\left(x;t_{\theta_{0}}^{c},P_{0}\right)\right\}=0.$$

On the other hand,

$$\begin{array}{c} \int \frac{\partial^{2}}{\partial \theta \partial t}m{\left[y,\theta ,t\right]}_{\theta =\theta_{0},t=t_{\theta_{0}}\left(P_{0}\right)}dP_{0}\left(y\right)=-\psi^{\prime }\left(\varphi^{\prime }\left(1\right)\right)\int \frac{\partial }{\partial \theta }\bar{g}\left(y,\theta_{0}\right)dP_{0}\left(y\right)\\ \;=-\int \frac{\partial }{\partial \theta }\bar{g}\left(y,\theta_{0}\right)dP_{0}\left(y\right), \end{array}$$

since $\psi^{\prime }\left(u\right)=\varphi^{\prime -1}\left(u\right)$. Taking into account that $t^{c}\left(\theta ,P_{0}\right)=t\left(\theta ,P_{0}\right)$ and $t(\theta ,P_{0})$ verifies

(A33) $$\int \frac{\partial }{\partial t}m\left(y,\theta ,t\left(\theta ,P_{0}\right)\right)dP_{0}\left(y\right)=0,$$

and the derivation with respect to θ yields

(A34) $$\int \frac{\partial^{2}}{\partial t\partial \theta }m\left(y,\theta ,t\left(\theta ,P_{0}\right)\right)dP_{0}\left(y\right)+\int \frac{\partial^{2}}{\partial^{2}t}m\left(y,\theta ,t\left(\theta ,P_{0}\right)\right)dP_{0}\left(y\right)\cdot \frac{\partial }{\partial \theta }t\left(\theta ,P_{0}\right)=0,$$

which implies

$$\begin{array}{c} \frac{\partial }{\partial \theta }t^{c}\left(\theta_{0},P_{0}\right)=\frac{\partial }{\partial \theta }t\left(\theta_{0},P_{0}\right)=-{\left\{\int \frac{\partial^{2}}{\partial^{2}t}m\left(y,\theta_{0},t\left(\theta_{0},P_{0}\right)\right)dP_{0}\left(y\right)\right\}}^{-1}\int \frac{\partial^{2}}{\partial t\partial \theta }m\left(y,\theta_{0},t\left(\theta_{0},P_{0}\right)\right)dP_{0}\left(y\right)\\ \;=-\varphi^{\prime \prime }\left(1\right){\left\{\int \bar{g}\left(y,\theta_{0}\right)\bar{g}{\left(y,\theta_{0}\right)}^{⊤}dP_{0}\left(y\right)\right\}}^{-1}\int \frac{\partial }{\partial \theta }\bar{g}\left(y,\theta_{0}\right)dP_{0}\left(y\right), \end{array}$$

because

(A35) $$\int \frac{\partial^{2}}{\partial^{2}t}m\left(y,\theta_{0},t\left(\theta_{0},P_{0}\right)\right)dP_{0}\left(y\right)=-\frac{1}{\varphi^{\prime \prime }\left(1\right)}\int \bar{g}\left(y,\theta_{0}\right)\bar{g}{\left(y,\theta_{0}\right)}^{⊤}dP_{0}\left(y\right).$$

Then, (A32) becomes

$$\begin{array}{c} {\left[\int \frac{\partial }{\partial \theta }\bar{g}\left(y,\theta_{0}\right)dP_{0}\left(y\right)\right]}^{⊤}\left[-\varphi^{\prime \prime }\left(1\right){\left\{\int \bar{g}\left(y,\theta_{0}\right)\bar{g}{\left(y,\theta_{0}\right)}^{⊤}dP_{0}\left(y\right)\right\}}^{-1}\int \frac{\partial }{\partial \theta }\bar{g}\left(y,\theta_{0}\right)dP_{0}\left(y\right)\mathrm{IF}\left(x;T^{c},P_{0}\right)\right.\\ \left.+\mathrm{IF}(x;t_{\theta_{0}}^{c},P_{0})\right]=0, \end{array}$$

and consequently,

(A36) $$\begin{array}{c} \mathrm{IF}\left(x;T^{c},P_{0}\right)\;\;=\;\;{\left\{{\left[\frac{\partial }{\partial \theta }\bar{g}\left(y,\theta_{0}\right)dP_{0}\left(y\right)\right]}^{⊤}{\left[\int \bar{g}\left(y,\theta_{0}\right)\bar{g}{\left(y,\theta_{0}\right)}^{⊤}dP_{0}\left(y\right)\right]}^{-1}\left[\frac{\partial }{\partial \theta }\bar{g}\left(y,\theta_{0}\right)dP_{0}\left(y\right)\right]\right\}}^{-1}\\ \;\cdot {\left[\frac{\partial }{\partial \theta }\bar{g}\left(y,\theta_{0}\right)dP_{0}\left(y\right)\right]}^{⊤}\frac{1}{\varphi^{\prime \prime }\left(1\right)}\mathrm{IF}\left(x;t_{\theta_{0}}^{c},P_{0}\right). \end{array}$$

□
